# Prime-Boost Immunization of Rabbits with HIV-1 gp120 Elicits Potent Neutralization Activity against a Primary Viral Isolate

**DOI:** 10.1371/journal.pone.0052732

**Published:** 2013-01-09

**Authors:** Kristin M. Narayan, Nitish Agrawal, Sean X. Du, Janelle E. Muranaka, Katherine Bauer, Daniel P. Leaman, Pham Phung, Kay Limoli, Helen Chen, Rebecca I. Boenig, Terri Wrin, Michael B. Zwick, Robert G. Whalen

**Affiliations:** 1 Department of Infectious Diseases, Maxygen, Inc., Redwood City, California, United States of America; 2 Altravax, Inc., Sunnyvale, California, United States of America; 3 Department of Immunology and Microbial Sciences, The Scripps Research Institute, La Jolla, California, United States of America; 4 Monogram Biosciences, Inc., San Francisco, California, United States of America; Boston College, United States of America

## Abstract

Development of a vaccine for HIV-1 requires a detailed understanding of the neutralizing antibody responses that can be experimentally elicited to difficult-to-neutralize primary isolates. Rabbits were immunized with the gp120 subunit of HIV-1 JR-CSF envelope (Env) using a DNA-prime protein-boost regimen. We analyzed five sera that showed potent autologous neutralizing activity (IC50s at ∼10^3^ to 10^4^ serum dilution) against pseudoviruses containing Env from the primary isolate JR-CSF but not from the related isolate JR-FL. Pseudoviruses were created by exchanging each variable and constant domain of JR-CSF gp120 with that of JR-FL or with mutations in putative N-glycosylation sites. The sera contained different neutralizing activities dependent on C3 and V5, C3 and V4, or V4 regions located on the glycan-rich outer domain of gp120. All sera showed enhanced neutralizing activity toward an Env variant that lacked a glycosylation site in V4. The JR-CSF gp120 epitopes recognized by the sera are generally distinct from those of several well characterized mAbs (targeting conserved sites on Env) or other type-specific responses (targeting V1, V2, or V3 variable regions). The activity of one serum requires specific glycans that are also important for 2G12 neutralization and this serum blocked the binding of 2G12 to gp120. Our findings show that different fine specificities can achieve potent neutralization of HIV-1, yet this strong activity does not result in improved breadth.

## Introduction

A major challenge in developing a protective vaccine for HIV-1 is the identification of an immunogen that can elicit potent and broad-spectrum neutralizing antibodies to primary isolates [1], [2]. Efforts to identify and characterize monoclonal antibodies (mAbs) from humans have provided important insights into the targets and molecular mechanisms of HIV-1 neutralization [3]–[13]. However, using this knowledge to rationally develop an effective vaccine continues to be difficult [14], thus highlighting the need for empirical approaches in HIV-1 vaccine research.

The envelope glycoprotein (Env) of HIV-1 forms functional spikes that mediate virus entry into host cells. Env engages the cellular receptor, CD4, which enhances the ability of Env to bind to the coreceptor, CCR5 or CXCR4 [15]. As a gp160 precursor, Env forms trimers and is extensively modified with high mannose residues at potential N-glycosylation sites (PNGS) [16]. The trimer is cleaved by furin leaving the extracellular gp120 and transmembrane gp41 subunits loosely associated and subject to further processing to display more complex glycans [17]. Glycosylation of native Env is rich in N-linked high-mannose residues, but also involves complex modified sugars and O-linked glycans [18]–[23]. Env biosynthesis [24], HIV-1 infectivity [25], pathogenesis [26], and escape from humoral immunity [16], [27], [28] are all affected by glycosylation as are the antigenic and immunogenic properties of Env [27], [29]–[36].

Eliciting neutralizing antibody to conserved epitopes on the gp120 subunit of native Env is extremely challenging. Thus, the CD4-binding site (CD4BS) is a target of broadly neutralizing mAbs such as b12 [37] and VRC01 [7], [8]. However, the CD4BS on native Env trimers is partially occluded by V1V2, V5 and/or proximal glycans [3], [8], [37]–[39], so many more CD4BS Abs including mAb b6 cannot neutralize primary isolates of HIV-1. V1V2 and V3 contain highly conserved residues, main chain elements and glycans that are recognized by broadly neutralizing mAbs like PG9/PG16 and PGT128, respectively [9], [40]–[42]. However, V1V2 and V3 also contain highly variable residues that can shield the more conserved regions. Some Abs to gp120 have only a limited capacity to neutralize because their epitopes are not fully revealed until receptor engagement, such as mAbs 17b [43] and X5 [44], [45] that target the bridging sheet of gp120, as well as mAbs 447-52D, 19b and F425-B4e8 (F425) that bind to the crown of V3 [46]. One broadly neutralizing mAb, 2G12, appears to bind exclusively to a cluster of high-mannose residues on the outer domain of gp120 [47]–[49]. However, 2G12 has a unique domain-swapped topology and it is unclear whether such an antibody can be re-elicited by design [50].

Many forms of Env have been evaluated as candidate vaccines to HIV-1. Monomeric gp120 typically elicits antibodies that neutralize only sensitive or lab-adapted strains with V3 sequences similar to that of the immunogen [4], [51]–[53]. Engineered gp120, gp140, or membrane-associated Env occasionally elicit neutralizing responses to V1 [54], V2 [55]–[57], variably exposed epitopes overlapping with receptor sites [58], [59], or other unidentified sites [51], [60], [61]. Virion-associated Env has elicited neutralizing antibodies of limited potency and breadth [62]–[65]. Attempts to elicit 2G12-like antibodies using synthetic glycoconjugates and hypermannosylated-yeast glycoproteins have shown that while the component glycans are immunogenic, the antibodies induced can neither neutralize primary virus nor block 2G12 binding to gp120 [21], [66]–[70]. Here, we show that strong autologous neutralization of the primary isolate JR-CSF can be elicited by prime-boost immunization with gp120. Because potent neutralization of resistant viruses such as JR-CSF [71] has not been readily achieved by immunization, understanding the neutralizing specificities elicited may assist in developing a prophylactic HIV-1 vaccine.

## Materials and Methods

### Plasmids

Full-length *env* genes from JR-CSF (AY669726) and JR-FL (AY669728) were cloned into the pMAmp expression vector described previously [52], [61], [72]. Domain-swap gp160 genes were generated using overlapping forward and reverse primers that bridge the JR-CSF-JR-FL boundary at each domain of interest. Overlap-extension PCR [73] was carried out using Platinum Pfx polymerase (Invitrogen). Two or more PCR fragments containing a region of overlap were synthesized in a first step, and then the amplicons were assembled in a second step. PNGS mutant genes were generated using mutagenic primers that change the targeted codons. Primers were purchased from Integrated DNA Technologies (Coralville, IA). Details of the oligonucleotide sequences, templates, and restriction sites used to generate each clone are available upon request. Ultracompetent XL-10 E. coli (Stratagene) were transformed with ligated DNA, colonies were expanded in liquid cultures, and plasmid DNAs were prepared using Qiagen QIAprep Spin Miniprep Kit. Plasmids were sequenced using a set of five primers to cover the entire gp160 open reading frame. Sequence contigs were generated and aligned (VectorNTI software) to confirm the correct sequence.

### Rabbit sera

Rabbit sera were obtained from immunizations carried out at Aldevron LLC (Fargo, ND) under animal use protocols 2-04-007-09-2005, 1-06-001-08-2007, and 1-07-005-10-2008 approved by the local Animal Care and Use Committee. Female New Zealand white rabbits were injected intramuscularly using the Inovio Twin Injector electroporation device with a total of 400 µg of a plasmid DNA construct encoding a codon-optimized JR-CSF gp120 DNA in the pMAmp vector. Animals were boosted with JR-CSF gp120 protein purified from CHO cells [72] and adjuvanted with AS02A [52], [61]. Five sera were chosen out of 24 rabbit sera from three different studies for a detailed characterization of the neutralizing specificities. These three studies examined the breadth and potency of neutralizing responses elicited by different Env immunogens, and the results will be published elsewhere. Rabbits from Study 32 (sera 3096 and 3099) and Study 41 (sera 3835 and 3844) were immunized with three injections of DNA on days 0, 28 and 56, boosted with protein on day 84, and a terminal bleed was collected on day 98. Rabbits from Study 14 (serum 1252) were immunized using DNA electroporation on days 0 and 28, boosted with protein on days 56, 84, 168, 252, and 336, and a terminal bleed was collected on day 350. Only the terminal bleeds were used in this study.

### Antibodies, CD4-IgG2, and human plasma

The monoclonal antibodies used in this work were obtained as follows: VRC01 [8], [12] from J. Mascola (Vaccine Research Center, NIH); b12 [37], b6 [38], and X5 [45] from D. Burton (The Scripps Research Institute); F425-B4e8 [74] from L. Cavacini (Dana Farber/Harvard Cancer Center); 2G12 [75] from Polymun (Klosterneuburg, Austria); and 2F5 and 4E10 [76] from the NIH AIDS Research and Reference Reagent Program (ARRRP). CD4-IgG2 [77] was provided by William Olsen of Progenics Pharmaceuticals (Tarrytown, NY). A broadly neutralizing human plasma, called Z23, was obtained by Monogram Biosciences from Zeptometrix (Buffalo, NY).

### Trofile™ and PhenoSense™ assays

JR-CSF and mutant *env* genes were cloned into the pCXAS-PXMX expression plasmid, and pseudoviruses were prepared as previously described [3], [78]. Briefly, the pCXAS-env plasmid and RTV1.F-lucP.CNDOΔU3, a replication-defective HIV-1 genomic vector that contains a luciferase expression cassette inserted within a deleted region of *env*, were co-transfected into 293T cell cultures. Virus-containing supernatants were harvested approximately 48 h after transfection. To determine infectivity and coreceptor tropisms of variant PSVs, CD4+/U87 cells expressing either CXCR4 or CCR5 were inoculated with PSV in a 96-well format. Successful infection leads to the production of luciferase in target cells. Coreceptor tropism was assessed by evaluating luciferase activity in lysed cells 72 h post-inoculation.

PhenoSense™ neutralization assays [3], [78] were performed by incubating pseudoviruses for 18 h at 37°C with ten 3-fold serial dilutions of serum, usually starting from a 1∶10 dilution, or mAb, starting at a concentration of 25 or 50 µg/ml. Virus infectivity was determined 72 h post-inoculation by measuring the amount of luciferase activity expressed in target cells. Neutralizing activity is reported as the mAb concentration (in µg/ml) or the reciprocal of the serum dilution (1/dilution) required to confer 50% inhibition of infection (IC50). The use of IC50 is appropriate because the neutralization curves are highly reproducible and the IC50 values can be accurately estimated.

### TZM-bl infectivity assay and production of PSVs with immature glycosylation

TZM-bl cells [79] were obtained from Dr. John C. Kappes, Dr. Xiaoyun Wu, and Tranzyme Inc. via the NIH ARRRP. Production of HIV-1 PSVs and TZM-bl neutralization assays were carried out as described previously [80]. To produce virus in the presence of kifunensine (kif), 25 µM kif (Sigma-Aldrich) was added to the 293T cell culture media immediately prior to transfection. The 293GnTI−/− cell line, a gift from the H.G. Khorana lab at MIT [81], was used to produce PSVs as follows. Cells were seeded in DMEM containing 5% heat-inactivated fetal bovine serum, 1× non-essential amino acids (NEAA), 1× sodium pyruvate as well as 50 µg/ml gentamycin, and incubated for 72 h prior to transfection. The 293GnTI−/− cells were cotransfected as described above in media consisting of DMEM, 1× NEAA, and 1× sodium pyruvate. After 5 h, the transfection media was replaced with expression media consisting of DMEM, 0.05% heat-inactivated fetal bovine serum, 1× NEAA, 1× sodium pyruvate, and 50 µg/ml gentamycin and incubation continued for 96 h prior to harvesting as described above for 293T cells. Neutralization of virus produced in 293T, 293T/kif, or 293GnTI−/− cells was assessed using TZM-bl cells.

### mAb Competition ELISA

Microtiter plate wells (flat-bottom; half-diameter, high protein binding, Corning) were coated overnight at 4°C with 30 ng JR-CSF gp120 in PBS. Biotinylated b12, 2G12 and VRC01 were produced using EZ-Link-sulfo-NHS-biotin reagent (Pierce) according to the manufacturer's instructions. The wells were washed four times with PBS containing 0.05% Tween 20 (PBST) and blocked for 1 h with 4% nonfat dry milk in PBST at 37°C. After four washes with PBST, 25 µl of sera (1∶10 dilution, 1∶5 titration) was added to each well and incubated for 30 min at 37°C, followed by an equal volume of biotinylated mAb at a constant concentration. The final concentrations of biotinylated Abs were previously determined to generate non-saturating binding signals. The plate was then incubated for 1.5 h at 37°C. Unbound antibody was removed by washing six times with PBST. Bound bio-mAb was detected using streptavidin-horseradish peroxidase conjugate (Jackson ImmunoResearch) diluted 1∶1000. Following incubation at room temperature for 1 h, the wells were washed six times, and developed using 50 µl tetramethylbenzidine (TMB) solution (Pierce), according to the manufacturer's instructions. After 10 min, wells containing TMB were quenched by adding 50 µl H_2_SO_4_ (2N) and the optical density at 450 nm was read on a microplate reader (Molecular Devices). Data were normalized to the maximal ELISA binding signal of the biotinylated mAb in the absence of competitor. Results were plotted as relative binding affinity using a four-parameter sigmoidal dose-response equation (variable slope) with GraphPad Prism Software 5.0. Each competitor was tested in duplicate.

## Results

### Autologous neutralizing sera from rabbits immunized with JR-CSF gp120 using a DNA-prime protein-boost protocol

**Figure 1:**
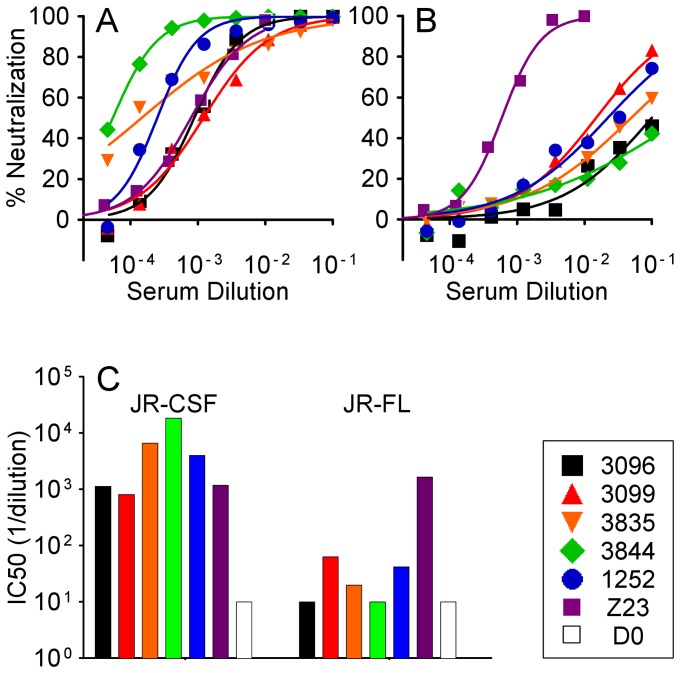
Neutralization activity of rabbit sera against JR-CSF (A) and JR-FL (B) pseudoviruses in the PhenoSense™ assay. A human plasma sample (Z23) is included for comparison. The x-axis in A and B represents the dilution of sera or plasma tested. (C) IC50 values (1/dilution) of all rabbit sera and the human plasma against JR-CSF and JR-FL pseudoviruses calculated from the curves in A and B. D0: Day 0 pre-immune rabbit serum.

We found that immunization of rabbits using a DNA vaccine encoding JR-CSF gp120 followed by a boost of sequence-matched gp120 protein can elicit neutralizing antisera against pseudoviruses carrying the JR-CSF Env (Figure 1 and Table 1) [52]. We chose five sera with potent autologous activity to identify the regions of Env that are targeted by neutralizing antibodies. These sera represent the highest JR-CSF neutralizing titers from twenty-four rabbits across three studies (Figure S1) with IC50 values in the PhenoSense™ assay ranging from 807 to 18,248 (Figure 1A and C, Table 1). We also tested neutralizing activity with an alternative assay using TZM-bl target cells (Table 2, “293T” column for JR-CSF) and still observed high IC50 values specific to the JR-CSF PSV. Serum 1252 showed more disparate results in the two assays with an IC50 of 3984 in the PhenoSense™ (Table 1) and 97 in the TZM-bl assay (Table 2).

**Table 1:** Comparison of neutralization activities (IC50) of rabbit sera against JR-CSF, JR-FL, and Tier I PSVs using the PhenoSense™ assay.

Serum	JR-CSF	JR-FL	SF162	NL4-3	BaL	6535
3096	1,128	<10	13,751	254	n.d.	n.d.
3099	807	63	84,702	537	n.d.	n.d.
3835	6,599	20	24,663	1,340	695	109
3844	18,248	<10	5,872	117	486	11
1252	3,984	42	5,472	3,377	227	146
Z23	1,172	1,649	59,053	7,196	5,054	1,086
Day 0	<10	<10	<10	<10	<10	<10

n.d., not determined.

**Table 2:** Comparison of neutralization activities (IC50) of rabbit sera against JR-CSF and JR-FL PSVs produced in 293T cells in the presence or absence of kifunensine (kif) or in 293GnTI−/− cells using the TZM-bl assay.

	JR-CSF IC50 (dilution^−1^)			JR-FL IC50 (dilution^−1^)		
Serum	293T	293T+kif	293 GnTI^−/−^	293T	293T+kif	293 GnTI^−/−^
3096	917	4,372	6192	<4	895	4
3099	298	6,614	5212	<4	1,755	14
3835	1,077	>10,240	>10,240	<10	960	<10
3844	13,011	>31,250	>31,250	<10	512	<10
1252	97	859	2704	<10	157	9

	JR-CSF IC50 (µg/ml)			JR-FL IC50 (µg/ml)		
mAb	293T	293T+kif	293 GnTI^−/−^	293T	293T+kif	293 GnTI^−/−^
2G12	2.10	0.99	1.10	9.30	0.31	1.19
b12	0.06	0.17	0.03	0.13	0.11	0.03

**Figure 2:**
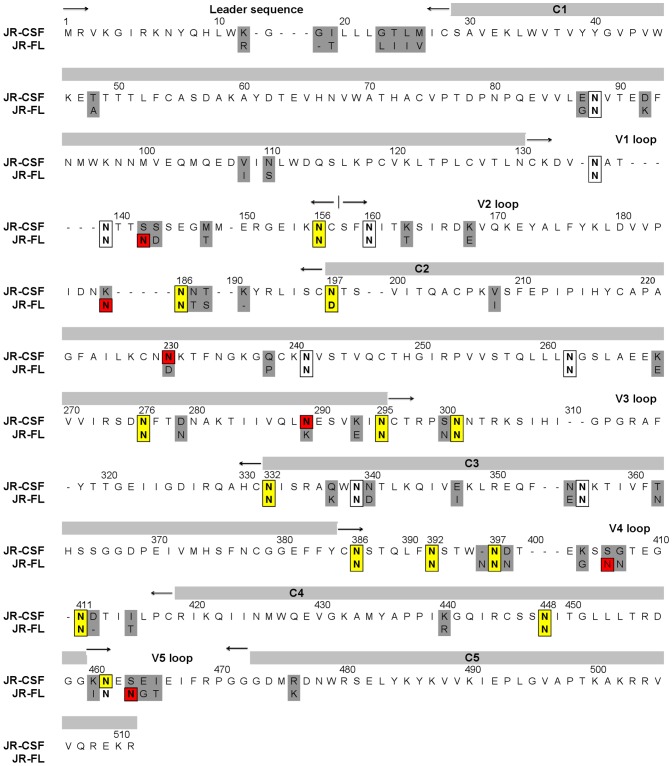
Amino acid sequence alignment of the JR-CSF and JR-FL gp120 proteins. Amino acid differences are highlighted by grey shading. The potential N-linked glycosylation sites (PNGS) are indicated as follows: PNGS targeted by mutagenesis (yellow boxes); non-targeted PNGS common to JR-CSF and JR-FL (empty boxes); and unique PNGS present only in JR-CSF or JR-FL (red boxes). The constant and variable regions of gp120 are indicated above the aligned sequences using thick grey shading and inward pointing arrows, respectively.

These five sera showed little or no neutralization of a PSV containing the JR-FL Env (Figure 1B and C, Table 1). JR-CSF and JR-FL are primary isolates of HIV-1 that were originally obtained from the same patient [82], and the two Envs share 92.5% amino acid identity (Figure 2). To further assess breadth of the activity, we tested the rabbit sera against several clade B pseudoviruses (Table 1). SF162 was strongly neutralized with some sera possessing IC50 values comparable to a broadly neutralizing human plasma (Z23 in Table 1). The rabbit sera were not as potent as Z23 against three other strains (6535, BaL, NL4-3). The other nineteen sera typically showed neutralizing activity against SF162 and NL4-3 (data not shown), irrespective of their potency against JR-CSF (Figure S1). These data indicate that immunization of JR-CSF gp120 using DNA priming followed by protein boosting elicits strong (autologous) neutralization activity of limited breadth.

### Domain swaps in V4, V5 and C3 disrupt autologous neutralization of JR-CSF

The serum antibodies that neutralize JR-CSF but not JR-FL presumably target regions in Env that differ between the two viruses. We therefore made a series of PSVs with chimeric Env proteins in which each domain of the JR-CSF gp120 sequence (C1, V1V2, C2, V3, C3, V4, C4, V5, and C5) was replaced with the corresponding domain of JR-FL (Figure 2). These chimeric “domain-swap” PSVs were tested for tropism and infectivity using the Trofile™ assay [83]. All PSVs were found to be R5 tropic and most were similar in infectivity to parental JR-CSF (data not shown).

**Figure 3:**
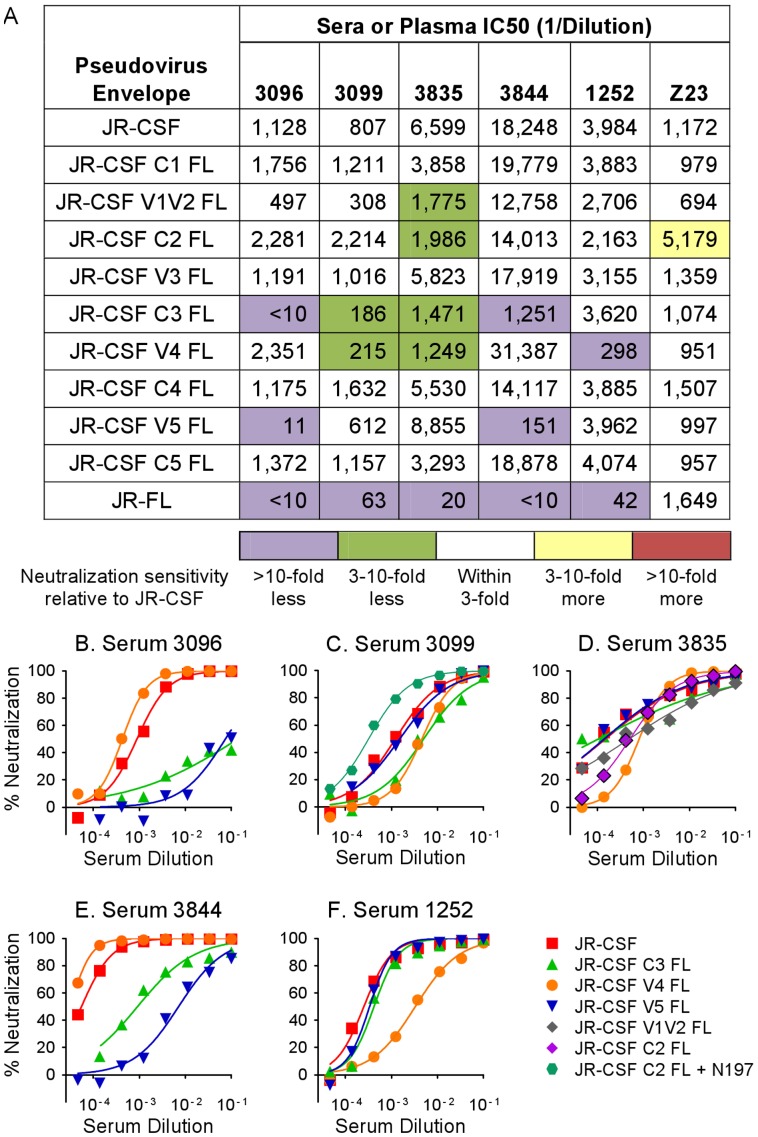
Rabbit sera contain neutralizing activities targeting multiple regions in JR-CSF gp120. (A) IC50 values (1/dilution) in the PhenoSense™ assay for rabbit sera and human plasma (Z23) against JR-CSF, JR-FL, and domain-swap pseudoviruses. The IC50 data are color-coded as described in the legend at the bottom to indicate the fold-change in neutralization sensitivity calculated as (IC50 JR-CSF)/(IC50 variant). (B–F) Neutralization curves for wild-type JR-CSF and those domain-swap pseudoviruses with altered sensitivity to rabbit sera (see text).

The neutralizing activities of sera 3096 and 3844 against JR-CSF substantially decreased after substitution of C3 or V5 with the corresponding JR-FL sequences (Figure 3A, B, and E). In contrast, the anti-JR-CSF activity of 3099 was diminished by swapping of C3 or V4 (Figure 3A and C). The potency of serum 3835 was reduced by swapping of V1V2, C2, C3, and C4, implicating multiple regions in the neutralizing epitopes targeted by this serum (Figure 3A and D). The neutralizing activity of serum 1252 was not impaired by swapping of C3, but it was reduced by the change of V4 (Figure 3A and F). The human plasma Z23 had similar activity against all domain-swap PSVs except for JR-CSF C2 FL, which showed modestly enhanced sensitivity to Z23. These data associate the autologous JR-CSF activity to C3 and V5 (sera 3096 and 3844), C3 and V4 with possible contributions from V1V2 or C2 (sera 3099 and 3835), or V4 alone (sera 1252) on gp120.

### Domain swaps in C2 of Env alter accessibility to the CD4BS

**Figure 4:**
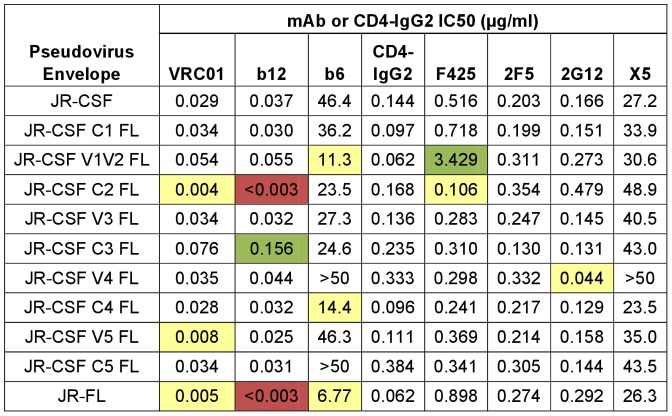
Domain swap of C2 alters pseudovirus sensitivity to mAbs. IC50 values (µg/ml) in the PhenoSense™ assay for mAbs and CD4-IgG2 against JR-CSF, JR-FL, and domain-swap pseudoviruses. The color-coding scheme is the same as in Figure 3A. * X5 neutralization was tested in the absence of CD4.

Incorporation of JR-FL sequences into the JR-CSF PSV could modify the conformation of the Env trimer and accessibility of neutralizing epitopes compared to wild-type JR-CSF. We therefore tested the neutralization sensitivity of the domain-swap PSVs to mAbs targeting the CD4BS (VRC01, b12, b6, and the immunoadhesin CD4-IgG2), the CD4i epitope (X5), the V3 loop (F425), the silent-face glycan cluster on gp120 (2G12), and the MPER region of gp41 (2F5). JR-FL was more sensitive to VRC01, b12, and b6 (Figure 4), which has been previously observed for VRC01 [7] and b12 [3], [7]. The sensitivity of domain-swapped PSVs to CD4-IgG2, 2F5, 2G12, and X5 was not much affected by any domain replacement, indicating that the domain swaps did not globally alter neutralization sensitivity of Env. Surprisingly, replacement of the C2 region increased sensitivity to VRC01, b12, and F425, suggesting that the JR-FL C2 contains sequences that expose CD4BS or V3 epitopes more than the JR-CSF C2. The presence of JR-FL V5 led to increased sensitivity to VRC01, possibly due to the close physical proximity of V5 to the heavy-light chain interface of VRC01 in structural models [8]. Exchange of V3, which only alters one residue (S300N, Figure 2), did not affect sensitivity to F425. The data obtained using domain-swap PSVs indicate that the specificities of the JR-CSF sera are clearly distinguishable from those of CD4-IgG2 or the mAbs tested.

### PNGS mutations in JR-CSF Env alter its sensitivity to autologous sera

**Figure 5:**
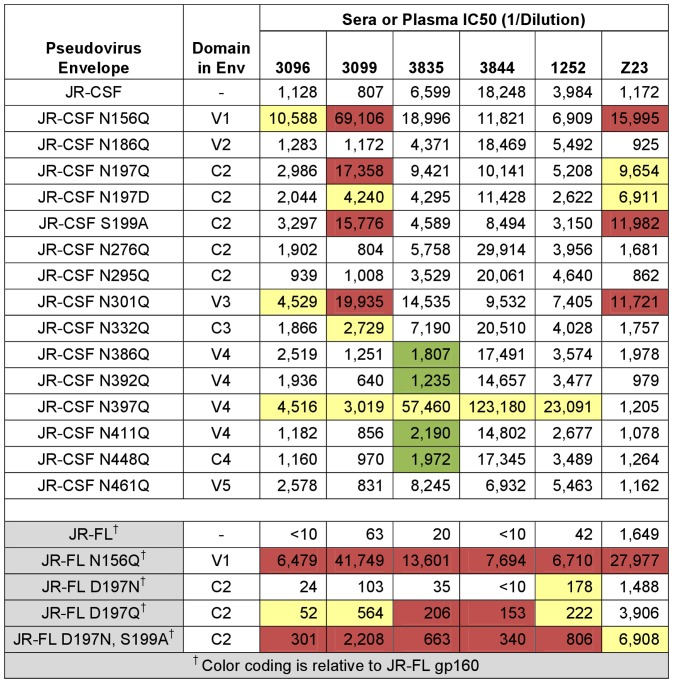
PNGS mutations modify the neutralizing activity of rabbit sera. IC50 values (1/dilution) in the PhenoSense™ assay for rabbit sera and human plasma (Z23) against pseudoviruses containing the indicated PNGS mutations. The color-coding scheme is the same as in Figure 3A; the fold-change values for pseudoviruses marked with a cross (†) are calculated relative to JR-FL.

Individual glycans can obscure neutralizing epitopes on Env while their removal (by mutation of the PNGS) can increase sensitivity of HIV-1 to neutralization [27], [29]–[35]. We targeted 13 PNGS in JR-CSF and two in JR-FL to further map the epitopes recognized by the rabbit sera (Figure 2 and Figure 5). In most cases, the canonical asparagine (N) was substituted with a glutamine (Q) in the consensus N-glycosylation sequon, NXS/T [84]. We also created additional mutants in which N was changed to aspartic acid (D) or the serine (S) was changed to alanine (A) to confirm whether the effect was due to loss of glycosylation or the amino acid substitution. PSVs formed by these mutated Envs were all R5-tropic but varied in infectivity compared to JR-CSF or JR-FL (data not shown). Notably, infectivity was substantially reduced for the mutant N156Q in both Env backgrounds and for D197Q in the JR-FL background. Infectivity was severely impaired by S158A, which disrupts a PNGS at N156 in both JR-CSF and JR-FL, and the JR-CSF S158A and JR-FL S158A PSVs were not further analyzed.

We next examined the neutralizing activity of the rabbit sera on the JR-CSF PSVs carrying PNGS mutations (Figure 5, top panel). PSVs with N156Q or N301Q increased sensitivity to serum 3096, while those with N to Q mutations at N156, N197, N301 or N332 were more sensitive to serum 3099 (Figure 5). This pattern of reactivity, particularly for 3099, was similar to Z23 human plasma. Three mutations that affected the same PNGS in JR-CSF (N197Q, N197D, and S199A) produced similar changes in sensitivity to the sera (Figure 5, top panel), suggesting that loss of glycosylation, and not the replacement of a particular amino acid, alters neutralization sensitivity. The JR-CSF N397Q PSV was hypersensitive to all five rabbit sera, but not to Z23. JR-CSF N397Q was the only PNGS mutation that altered neutralization by sera 3844 or 1252. Serum 3835 showed a unique profile where loss of a PNGS actually reduced sensitivity against the JR-CSF mutants N386Q, N392Q, N411Q, and N448Q, indicating that the antibodies in this serum may recognize a glycan epitope involving these residues.

The PNGS at positions 156 and 197 are conserved in 94% and 98%, respectively, of Env sequences in the Los Alamos National Laboratory database (www.hiv.lanl.gov; accessed on June 15, 2011.). Loss of glycosylation at these positions has previously been shown to alter PSV sensitivity to neutralizing antibodies of different specificities [30], [31], [36]. We therefore studied how mutants at 156 and 197 affect neutralization by the sera in a JR-FL background (Figure 5, bottom panel). Mutation of the PNGS at 156 in JR-FL substantially increased sensitivity of the PSV to all five rabbit sera and Z23 likely due to a loss of glycosylation at this site that exposed epitopes that are typically shielded. JR-FL Env is unusual in that it does not have a PNGS at position 197; rather, aspartic acid (D) replaces the asparagine (N) at this position (Figure 2). We evaluated the effect of adding a glycan at this site (JR-FL D197N) or incorporating different amino acids (JR-FL D197Q and JR-FL D197N, S199A) in the sensitivity of JR-FL to the JR-CSF sera (Figure 5). Adding a PNGS at position 197 had little or no effect on neutralization compared to JR-FL, but introducing different other amino acids at this position did change sensitivity to the rabbit sera and Z23. These data suggest that the aspartic acid at position 197 of JR-FL is critical for access of Ab to this site in the absence of glycosylation.

### PNGS mutations indicate commonalities between the epitopes of mAbs and rabbit sera

**Figure 6:**
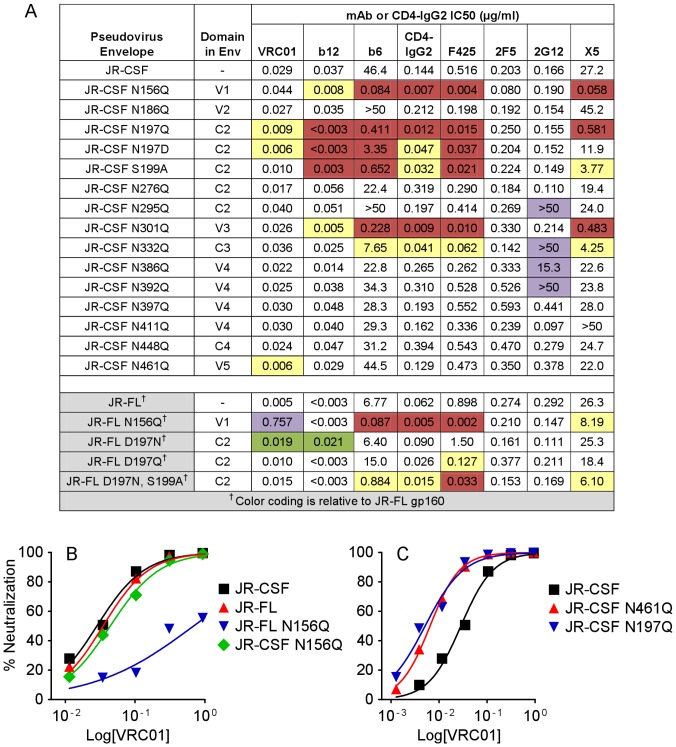
PNGS mutations demonstrate that similar PNGS are targeted by rabbit sera and mAbs. (A) IC50 values (µg/ml) in the PhenoSense™ assay for mAbs and CD4-IgG2 against pseudoviruses containing the indicated PNGS mutations. The color-coding scheme is the same as in Figure 3A; the fold-change for pseudoviruses marked with a cross (†) is calculated relative to JR-FL. *X5 neutralization was tested in the absence of CD4. (B and C) Neutralization curves showing the activity of VRC01 against JR-CSF, JR-FL, and the indicated PNGS mutants.

We evaluated each glycosylation mutant PSV for its sensitivity to VRC01, b12, b6, F425, X5, and CD4-IgG2 to compare the resulting neutralization profiles (Figure 6A, top panel) to those given by the rabbit sera. The sensitivity of the JR-CSF PSV to VRC01 was increased approximately 3- and 5-fold by the N197Q mutation at the base of V1V2 and the N461Q change in V5, respectively (Figure 6A and C). Mutations at N156, N197 and N301 hypersensitized JR-CSF PSV to b12, consistent with previous findings [28], [30], [31]. Although b6 does not neutralize JR-CSF at less than 50 µg/ml, loss of glycosylation at N156, N197 and N301, and to a lesser extent N332, greatly increased sensitivity of JR-CSF to this mAb. These PNGS mutations also increased sensitivity toward CD4-IgG2, F425 and X5, indicating that the glycans at N156, N197, N301 as well as N332 appear to protect JR-CSF PSV against neutralization by antibodies of multiple specificities. Sera 3099, Z23, and to some extent sera 3096, showed similar neutralization profiles against the same PNGS mutant PSVs (Figure 5), suggesting that the sera contain specificities similar to mAbs with limited neutralization capacity, such as b6, F425, or X5. Mutations at the PNGS sites N295, N332, N386, and N392 disrupted the neutralizing activity of 2G12 (Figure 6A), in agreement with previous studies [47], [48], [85]. Interestingly, mutation of PNGS N386 and N392 in JR-CSF also disrupted the neutralizing activity of sera 3835 (Figure 5). Finally, none of these PSVs showed a substantial change in sensitivity to the gp41 MPER mAb 2F5, suggesting that they were not globally altered in their reactivity with neutralizing antibodies.

We also studied the neutralization of JR-FL PSVs with mutated PNGS by the mAbs and CD4-IgG2 (Figure 6A, bottom panel). We found that JR-FL N156Q was 150-fold less sensitive to VRC01 than wild-type JR-FL while JR-CSF carrying the same mutation was only slightly less sensitive to VRC01 (Figure 6A and B); both mutant pseudoviruses were still hypersensitive to other CD4BS mAbs. Although VRC01 is relatively insensitive to substitutions in V1V2 compared to many other CD4BS mAbs [86], our results indicate that mutations in the V2 loop can diminish the potency of VRC01 considerably, at least in certain Env backgrounds (e.g., N156Q in JR-FL). However, because VRC01 is so potent against wild-type JR-FL, it still effectively neutralizes the N156Q mutant (IC50 values of 0.005 and 0.8 µg/ml, respectively, Figure 6A). Addition of a PNGS to JR-FL at amino acid 197 (JR-FL D197N, Figure 6A) decreased sensitivity of JR-FL to VRC01 and b12 roughly to JR-CSF levels, but sensitivity to b6 or CD4-IgG2 was not altered. This finding, along with the increased sensitivity of the JR-CSF C2 FL domain swap PSV to VRC01 and b12 (Figure 4) indicates that lack of a PNGS at 197 in the C2 domain contributes to the increased sensitivity of JR-FL to VRC01 and b12. Additional mutations in JR-FL around position 197, including D197Q and D197N/S199A, showed modest changes in sensitivity to b6, CD4-IgG2, F425, and X5. These data suggest that the presence or absence of glycans at positions 156 and 197 can play a pivotal role in protecting or exposing neutralizing epitopes overlapping and surrounding the CD4BS in a manner that depends on the Env background and the identity of the residue used to disrupt the PNGS.

### Immature glycosylation of Env increases sensitivity to autologous sera

Our results showed that specific glycans on JR-CSF gp120 can modulate the neutralizing activity of autologous rabbit sera, particularly with serum 3835 (Figure 5). The extent of glycan processing could also affect sensitivity of wild-type JR-CSF to these sera. We therefore tested the neutralization sensitivity of the JR-CSF PSV produced under two different conditions: (i) in the presence of the glycosidase inhibitor, kifunensine (kif), which leads to homogeneous, immature glycan structures composed of Man_9_GlcNAc_2_ [35], [85]; and (ii) in 293S cells that are deficient in N-acetylglucosaminyltransferase I (GnTI−/−), resulting in partially trimmed, but still immature Man_5_GlcNAc_2_ and higher order oligomannose structures [35], [87]. The PSVs produced in the presence of kif or in 293GnTI−/− cells showed increased sensitivity to all five rabbit sera but not to normal rabbit sera (data not shown) while also roughly preserving the rank order in serum potencies (Table 2). In contrast, sensitivity to the CD4BS mAb b12 and to the high-mannose dependent mAb 2G12 [47], [48], [75] was less affected by virion production using kif or GnTI−/− cells (Table 2). These latter results are largely consistent with previous data except that others had found 2G12 potency to be slightly more enhanced with kif-treatment than we observe with JR-CSF [35]. The heterologous JR-FL PSV became markedly sensitive to the JR-CSF-specific rabbit sera when produced in kif-treated cells but not when produced in GnTI−/− cells. However, even with kif-treatment, JR-FL is still not as sensitive as JR-CSF and the rank-order in serum potency is different for the two isolates (Table 2). Thus, while it is possible that kif-modified PSV has become sensitive to specificities in the rabbit sera (e.g., b6-like antibodies) that are different from the autologous JR-CSF-specific ones, it is also possible that the accessibility on Env to the JR-CSF-specific antibodies is regulated at least in part by the presence (or absence) of hybrid or complex glycan.

### Autologous neutralizing sera blocks 2G12 and CD4BS mAb binding to gp120

**Figure 7:**
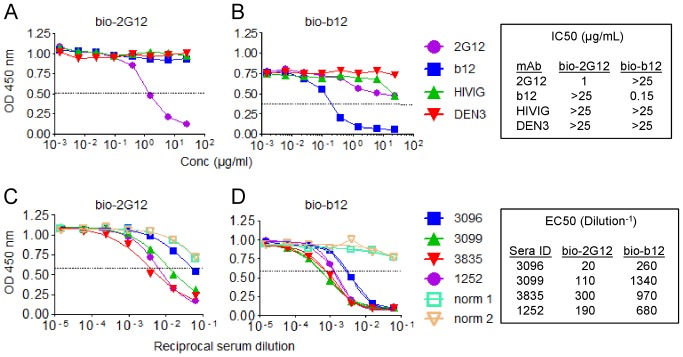
Rabbit sera block binding of mAbs to gp120. Competition ELISA using various mAbs and sera to block binding of biotinylated (bio) 2G12 (A, C) and b12 (B, D) to immobilized JR-CSF gp120. Two normal rabbit sera are used as non-immune controls. The results shown are representative of three independent experiments. The dashed horizontal line (—) corresponds to the half-maximal signal (EC50). HIVIG: HIV immune globulin provided by J. Mascola (VRC); DEN3: a mAb to dengue virus envelope provided by D. Burton (TSRI).

We tested whether the rabbit sera could affect the binding of several broadly neutralizing mAbs or CD4-IgG2 to JR-CSF gp120 (Figure 7). Each serum efficiently blocked the binding of VRC01, b12 and CD4-IgG2 (Figure 7B and D; data not shown), consistent with previous studies showing that polyclonal sera raised to gp120 can block b12 binding [88], [89]. Because the rabbit sera are polyclonal, it is unknown whether the competing antibodies are neutralizing, non-neutralizing or whether they bind to regions of Env that overlap with the CD4BS or obscure the CD4BS by another mechanism. Surprisingly, we also found that the rabbit sera effectively inhibited 2G12 binding to gp120 (Figure 7A and C); this was particularly strong with serum 3835 that showed features of glycan-dependent neutralization (Figure 5). It is noteworthy that serum 3835 was also the only serum with a non-sigmoidal dose response curve in neutralization assays with JR-CSF (Figure 1A), again suggesting that the neutralizing activities in this serum are distinct. The competition ELISA data indicate that antibodies elicited by immunization of rabbits using a JR-CSF gp120 DNA-prime protein-boost regimen can target epitopes that are either similar to, overlap with, or regulate access to the glycan epitope of the broadly neutralizing 2G12 mAb.

## Discussion

HIV-1 immunogens typically fail to elicit neutralizing antibody responses to panels of primary isolates [3], [90]–[92]. However, such panels often give little information on the fine specificities of the elicited antibodies that can neutralize a particular isolate. In this study, we used pseudoviruses carrying a number of variant Env proteins to investigate the specificity of rabbit sera that showed strong autologous activity against JR-CSF. The results showed that the C3 and V5, C3 and V4, or V4 regions of Env and specific glycans are largely responsible for this neutralization activity. Antibodies targeting regions in the outer domain of Env have been documented in other studies. The C3, V4, and V5 regions have recently been shown to participate in autologous neutralizing antibody responses that appear during early clade C HIV-1 infections [93], [94]. Binding antibodies to V4 or V5 have been experimentally elicited as evidenced by serum reactivity using immobilized peptides [63], [88], [89], [95], [96], but it is unknown if these are neutralizing antibodies. To our knowledge, this is the first demonstration that neutralizing antibodies can be elicited to these epitopes on a primary isolate by experimental immunization.

Although JR-CSF is a primary isolate [82] that is relatively resistant to neutralization [71], we find that potent inhibition of a JR-CSF pseudovirus can be obtained when the humoral response is directed to C3 and V5, C3 and V4, or V4 regions located on the glycan-rich outer domain of gp120. There is no statistical difference in the autologous JR-CSF neutralization between the immunization protocols used for rabbits in Studies 32 and 41 (three DNA primes followed by one protein boost) versus Study 14 (two DNA primes followed by five protein boosts, data not shown). Both protocols resulted in sera with IC50s against a JR-CSF pseudovirus of at least 10^3^. We typically observe a wide range of IC50s against JR-CSF within a group of sera from rabbits immunized using the same conditions, but we do not understand why some rabbits elicit high titers while others show little response. The strong neutralization of JR-CSF observed here using prime-boost immunization has not previously been described. Rabbit serum 3844 was particularly strong and gave an IC50 of over 18,000 and 13,000 against JR-CSF when measured independently in two different systems (the PhenoSense and TZM-bl assays, respectively). If the neutralizing antibody specificities in the undiluted rabbit serum were present at between 10–1,000 µg/ml [11], [97], these would have an IC50 of between 0.55–76 ng/ml, which is far lower than that of broadly neutralizing mAbs studied here. In other rabbit studies [88], [98], a prime-boost protocol elicited antisera that neutralized JR-FL in the TZM-bl assay with somewhat lower titers (i.e., up to 1∶160) but did not neutralize JR-CSF and were of limited breadth. It is therefore possible to elicit potent neutralization against primary isolates as demonstrated here and elsewhere, and the development of a consistent breadth of neutralization is the major obstacle to a workable vaccine concept.

Overall, the neutralizing specificities of the rabbit sera are distinct from those of well-studied mAbs, CD4-IgG2, or a broadly neutralizing human plasma (Z23). However, use of the variant PSVs shows that some sera target similar regions of Env as mAbs or CD4-IgG2. For example, PNGS mutations at N156, N197, N301, and N332 increased sensitivity to sera 3099, as well as to b6, CD4-IgG2, F425, and X5. One possibility is that serum 3099 contains antibodies to the CD4BS with specificities similar to those of b6 or F425, as these antibodies can only neutralize when glycans are removed that would otherwise protect the CD4BS and V3, respectively. In support of this explanation is the observation that JR-FL N156Q (but not wild-type JR-FL) is hypersensitive to the rabbit sera as well as to b6 and F425. In contrast, sera 1252, 3835, and 3844 are highly active against wild-type JR-CSF but are not more potent against JR-CSF N156Q, suggesting that neutralizing antibodies directed to V3/CD4BS/CD4i are absent in these sera and/or that the C3/V4-, C3/V5- and V4-dependent antibodies dominate or mask the neutralizing activity of antibodies like F425, b6 or X5. Mutation of two glycans that comprise the 2G12 epitope (N386 and N392) also diminished the neutralizing activity of rabbit serum 3835, suggesting an overlap between the neutralizing epitopes.

We mapped the Env domains and PNGS that altered the activity of the rabbit sera or select mAbs onto the gp120 core structure (Figure S2C) including N386 and N392, which are involved in the activity of serum 3835 and 2G12. Since the 2G12 site is preserved on outer domain constructs of gp120 and is distal to subunit interfaces, it is possible that antibodies that compete with 2G12 on monomeric gp120 would also bind to native trimers and neutralize HIV-1 [99]. The potency of all five rabbit sera was enhanced by mutation of a PNGS at position 397. Residue N397 follows the β18 strand in C3 just N-terminal to V4 of gp120 (Figure S2A and B). Thus, glycosylation at this residue appears able to at least partially occlude epitopes involving C3, V4 and V5. The VRC01 epitope on the structural map is largely distinct from the regions targeted by rabbit sera; however, elements of C2, C3 and V5 could overlap between these neutralizing epitopes as well (Figure S2C) [8].

The rabbit sera interfere with binding of 2G12, VRC01, b12 and CD4-IgG2 to gp120 in competition ELISA experiments. In particular, 2G12 is in a unique antibody competition class and no other immunized sera, and even very few HIV-1 patient sera, have been shown to block 2G12 binding to monomeric gp120 [66]–[70], [75], [89], [100], [101]. Intriguingly, several new “PGT” mAbs have recently been described with epitopes at the base of V3 that overlap the 2G12 site and show even greater breadth and potency of neutralization [11]. The immunogenicity of glycans on the outer face of gp120 in the present immunization study is a significant milestone, and we suggest that competition studies using antisera against 2G12 and VRC01 can be of value for gauging progress towards eliciting similar or overlapping specificities.

It is important to understand how sera from experimental immunizations of animals or humans can neutralize primary HIV-1 isolates in order to develop improved vaccines. We have obtained some insight on the regions of gp120 that are targets of a neutralizing response to the primary isolate JR-CSF. Whether such autologous neutralizing specificities can be matured by specific immunization strategies into more cross-reactive antibodies remains an important and open question.

## Supporting Information

Figure S1 **Prime-boost immunization using JR-CSF gp120 elicits a range of JR-CSF neutralizing activities.** IC50 values (1/dilution) of 24 sera from rabbits immunized with a JR-CSF gp120 DNA vaccine followed by JR-CSF gp120 protein are shown. The sera are from three immunization studies (Study 32, Study 41, and Study 14). The detailed immunization regime for each study can be found in the Materials and Methods. The geometric mean titer against JRCSF for each group is represented as a line. No statistically significant difference between the groups was observed. Five sera that represented the highest IC50 against JR-CSF within each group were selected for characterization of neutralizing activity using domain swap and glycovariant pseudoviruses. These sera are indicated with a red data point with the rabbit number adjacent to it (3096, 3099, 3835, 3844, and 1252).
(TIF)

Figure S2 **Regions of gp120 targeted by rabbit sera are distinct but show some overlap with mAbs.** The results of this study are represented visually using a model of the crystal structure of JR-FL gp120 core [102], shown here in the absence of bound CD4 and X5 antibody Fab fragment (pdb: 2B4C). (A) Positions of the asparagine residues in the 13 PNGS targeted in this study. Gp120 is shown in ribbon representation and the Asn side chains of each PNGS are shown in space-filling representation (blue). Regions of gp120 are color coded as follows: V1V2 stem (purple), V3 (fire red), V4 (orange) and V5 (cyan). Residues N156 and N186 are missing in the structure that lacks V1V2, so these are added but are not necessarily to scale. Position 197 is indicated and is part of a PNGS in JR-CSF, but is aspartic acid and not a PNGS in JR-FL. The black arrow indicates the approximate approach of CD4 to the CD4BS. (B) Regions of gp120 used for the domain-swap PSVs. The various domains are colored in ribbon representation as follows: C1 (grey), V1V2 stem (purple) C2 (yellow), V3 (red), C3 (green), V4 (orange), C4 (pink), V5 (cyan) and C5 (brown). Residues that differ between JR-FL and JR-CSF in the domains where substitution affected serum neutralization (C3, V4 and V5) are shown in space filling representation (grey). (C) Difference maps highlighting residues on the structure of gp120 whose substitution significantly affected neutralization of JR-CSF by each serum or mAb. Residues colored in green and red are those whose mutations lead to increased or decreased neutralization sensitivity of JR-CSF, respectively. The top four panels (left to right) and the bottom left panel show the difference maps for serum 3096 (C3 and V5); serum 3099 (C3 and V4); serum 3835 (C3 and V4); serum 3844 (C3 and V5); and serum 1252 (V4). The bottom panel labeled “Rabbit composite” shows a composite image that superimposes the maps of all five rabbit sera. The panel labeled “2G12” is a difference map using the data from Figure 5 and Figure 6. Asterisks (*) indicate those residues whose alteration reduces neutralization by both 2G12 and rabbit serum 3835. The bottom right panel labeled “VRC01” highlights the area on the CD4BS of gp120 that is targeted by VRC01 (dark gray). Molecular model images were generated using PyMOL software.
(TIF)
