# Supplementary figures and images for: Prime-Boost Immunization of Rabbits with HIV-1 gp120 Elicits Potent Neutralization Activity against a Primary Viral Isolate
Source: PLoS One. 2013 Jan 9;8(1):e52732. doi: 10.1371/journal.pone.0052732 (PMC3541383; doi:10.1371/journal.pone.0052732)

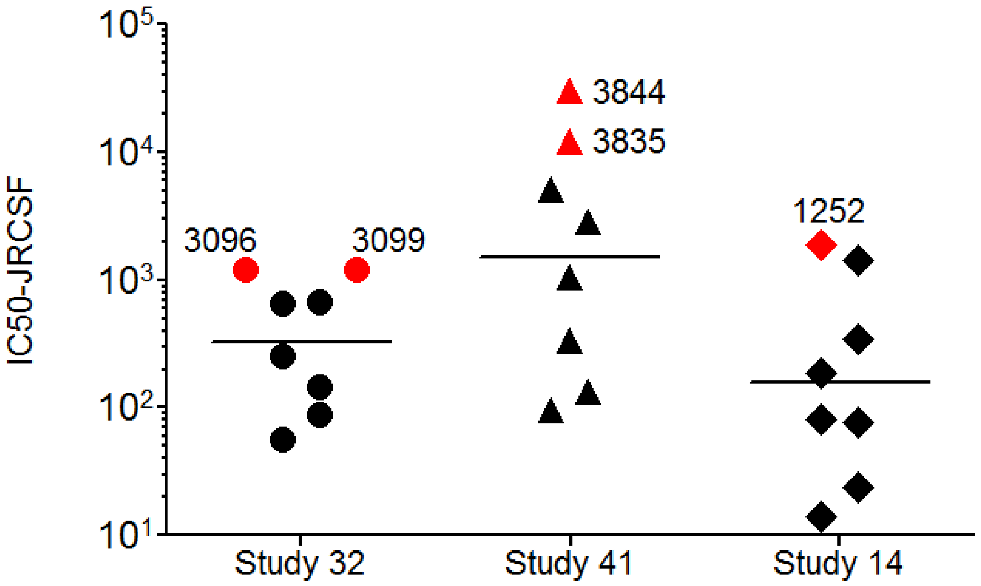

Supplement: Figure S1 — Prime-boost immunization using JR-CSF gp120 elicits a range of JR-CSF neutralizing activities. IC50 values (1/dilution) of 24 sera from rabbits immunized with a JR-CSF gp120 DNA vaccine followed by JR-CSF gp120 protein are shown. The sera are from three immunization studies (Study 32, Study 41, and Study 14). The detailed immunization regime for each study can be found in the Materials and Methods. The geometric mean titer against JRCSF for each group is represented as a line. No statistically significant difference between the groups was observed. Five sera that represented the highest IC50 against JR-CSF within each group were selected for characterization of neutralizing activity using domain swap and glycovariant pseudoviruses. These sera are indicated with a red data point with the rabbit number adjacent to it (3096, 3099, 3835, 3844, and 1252). (TIF) [file pone.0052732.s001.tif]

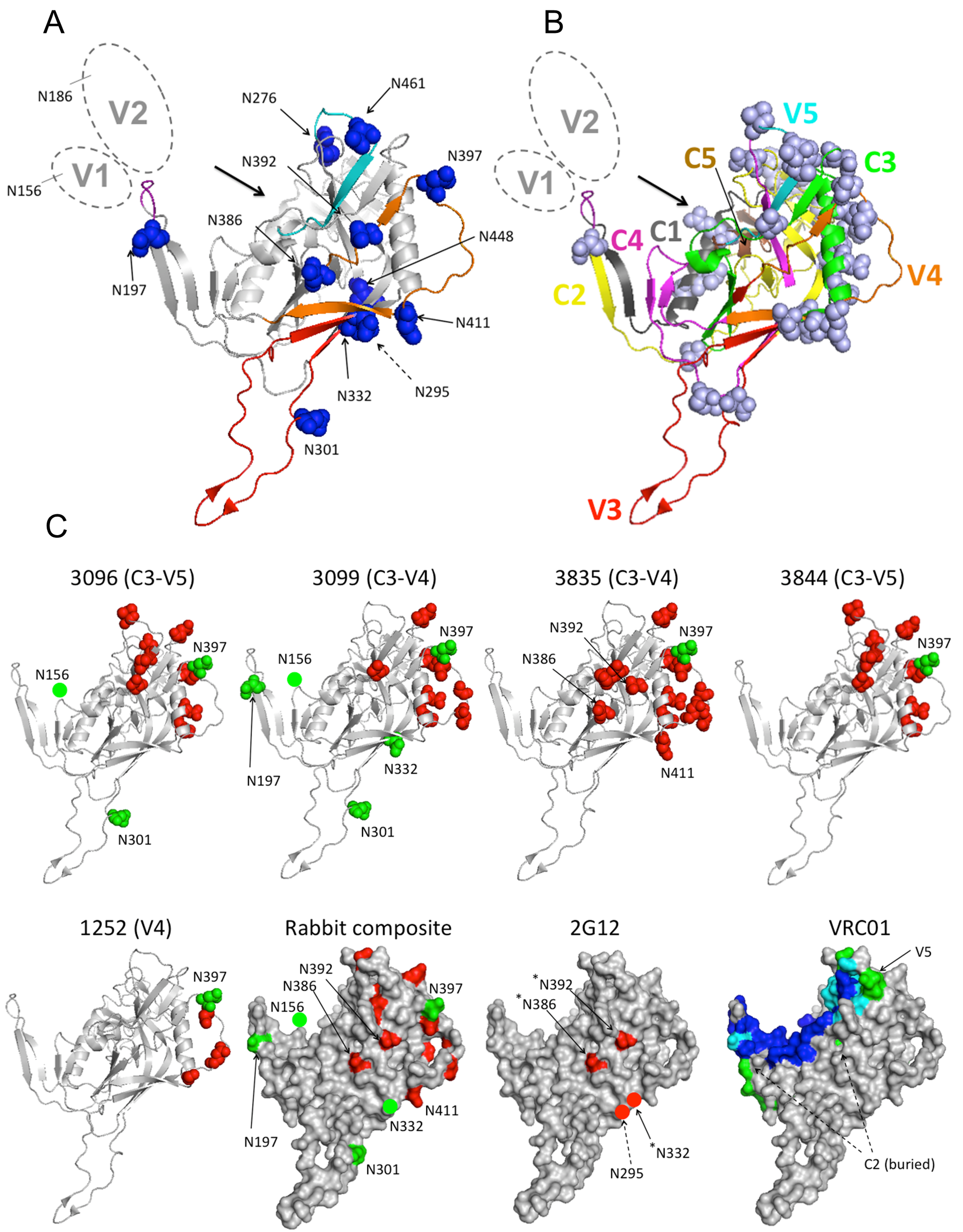

Supplement: Figure S2 — Regions of gp120 targeted by rabbit sera are distinct but show some overlap with mAbs. The results of this study are represented visually using a model of the crystal structure of JR-FL gp120 core [102], shown here in the absence of bound CD4 and X5 antibody Fab fragment (pdb: 2B4C). (A) Positions of the asparagine residues in the 13 PNGS targeted in this study. Gp120 is shown in ribbon representation and the Asn side chains of each PNGS are shown in space-filling representation (blue). Regions of gp120 are color coded as follows: V1V2 stem (purple), V3 (fire red), V4 (orange) and V5 (cyan). Residues N156 and N186 are missing in the structure that lacks V1V2, so these are added but are not necessarily to scale. Position 197 is indicated and is part of a PNGS in JR-CSF, but is aspartic acid and not a PNGS in JR-FL. The black arrow indicates the approximate approach of CD4 to the CD4BS. (B) Regions of gp120 used for the domain-swap PSVs. The various domains are colored in ribbon representation as follows: C1 (grey), V1V2 stem (purple) C2 (yellow), V3 (red), C3 (green), V4 (orange), C4 (pink), V5 (cyan) and C5 (brown). Residues that differ between JR-FL and JR-CSF in the domains where substitution affected serum neutralization (C3, V4 and V5) are shown in space filling representation (grey). (C) Difference maps highlighting residues on the structure of gp120 whose substitution significantly affected neutralization of JR-CSF by each serum or mAb. Residues colored in green and red are those whose mutations lead to increased or decreased neutralization sensitivity of JR-CSF, respectively. The top four panels (left to right) and the bottom left panel show the difference maps for serum 3096 (C3 and V5); serum 3099 (C3 and V4); serum 3835 (C3 and V4); serum 3844 (C3 and V5); and serum 1252 (V4). The bottom panel labeled “Rabbit composite” shows a composite image that superimposes the maps of all five rabbit sera. The panel labeled “2G12” is a difference map using the data fr [file pone.0052732.s002.TIF]
